# Uncovering heterogeneous interactions in online commercial networks

**DOI:** 10.1038/s41598-017-17410-1

**Published:** 2017-12-08

**Authors:** Fangfeng Zhang, An Zeng, Bowen Ma, Ying Fan, Zengru Di

**Affiliations:** 1 School of Information, Beijing Wuzi University, Beijing, 101149 PR China; 2 School of System Science, Beijing Normal University, Beijing, 100875 PR China; 3 Highland school, Warrenton, VA 20186 USA

## Abstract

With the rapid development of Internet, the research on online commercial networks has become crucial for filtering out irrelevant information for users and predicting their future interest. The common methods for understanding such typical user-item networks are mainly projecting them to unipartite ones with only positive ratings, which may result in losing a large amount of information. In this paper, we propose a novel approach to construct a signed unipartite network with heterogeneous interactions (i.e. positive or negative) between users from the original bipartite network. Based on the signed similarity, we carry out the percolation analysis on this signed unipartite network, which reveals a phase transition phenomenon. The statistical features of the giant component consisting of the positive and negative interactions are investigated respectively. Finally, the roles of the negative links and weak ties are revealed by adding them back to the giant component. This work not only deepens our understanding of the online commercial networks, but also has potential applications in the design of recommendation algorithms.

## Introduction

As an important type of complex network, bipartite network is an ubiquitous model describing a wide variety of phenomena in real life ranging from scientific publication systems (e.g. author-paper networks) to online commercial systems (e.g. user-item networks)^1–3^. The rapid development of information technology and Internet makes it necessary to investigate the structure and function of online user-item bipartite networks. The research on online commercial networks, as portrayed by user-item bipartite networks, is of great significance in not only filtering out irrelevant information for users but also predicting their future interest.

The analysis on bipartite networks is commonly conducted by projecting them to unipartite ones, e.g. projecting author-paper bipartite network to scientific collaboration network^4–6^. In the case of online commercial networks, such user-item networks are always projected to the networks between users, with a link representing two users have rated at least one common item^7,8^. As far as online commercial networks are concerned, the common approaches^1,2^ are mainly deleting dislike rating information and then projecting them to unipartite ones, which may result in losing a large amount of information. Many other real networks also contain signs (positive or negative) which includes a prevailing type of relations in social, biological and information systems, such as support or opposition, agreement or disagreement, and excitation or inhibition etc. Study of signed network thus plays an important role in accurately understanding complex systems and their applications. In online commercial systems, one can obtain a heterogeneous user networks with signed interactions by projecting online user-item bipartite network with both like ratings and dislike ratings information. Such signed network can better characterize the relation between users. Based on the heterogeneous interactions, the information filtering and personal recommendation will be more efficient^9^. Therefore, the research on signed online social networks is of both theoretical and practical values.

The related research works in signed networks in the context of social science have been summarized in a review article^10^. There are two basic theories concerning the signed networks: structural balance theory and status theory. Structural balance was first proposed by sociologists Heider^11^, which divides relationship between peoples into two types (i.e. positive or negative), and describes evolution of the relationship type by empirical analysis. After that, Cartwright and Harary^12^ presented a model of structural balance in mathematical positive and negative symbols. Weak structural balance theory on a wider constraint conditions was put forward by Davis^13^. Status theory, firstly brought out by Leskovec, Kleinberg and etc.^14^, is suitable for directed networks. They believed that the sign of one link was decided by the different status of the two nodes, that was, positive link was only established from nodes to other nodes with higher status. In particular, many studies have been devoted to studying the features of signed networks’ adjacency matrixes. Bonacich *et al*.^15^ represented the undirected signed network as an adjacency matrix, and properties of adjacency matrix with structural balance were discussed. Other works include directed signed networks extracted from Slashdot Zoo by Kunegis *et al*.^16^ and adjacency matrices of gene regulation on conditions of restrictive Boolean networks^17^. A number of research works aimed at identifying structure balance (especially in social systems) over the years, the most prominent measurement includes ratio of balance triangles to all triangles. Kunegis *et al*.^16^ called it signed clustering coefficient and further expanded it to directed unweighted signed networks by adding directional constraints. Leskovec *et al*.^9^ found structure balance in local area was much stronger than in global. In social systems, Bonacich *et al*.^15^ analyzed the structure properties and identified important nodes of monks’ network in a monastery. Leskovec, Kleinberg *et al*.^14^ discussed how the positive and negative interactions affected the structure of online social networks by several empirical analysis of status theory. Szell *et al*.^18^ discovered the different statistical characteristics between positive and negative edges through constructing triangle structures in large-scale online games social networks.

Despite that several signed networks have been investigated, we still lack the systematic understanding of the comprehensive features of heterogeneous interaction networks. There are still challenges and difficulty in uncovering heterogeneous interaction in online commercial networks. In this paper, we refer to heterogeneity as signs of links (i.e. positive or negative). However, the signs of users’ interactions are usually hidden. Additionally, there is still no universally agreed-on definition of negative interactions in network construction, especially when projecting bipartite networks to unipartite ones. During the projection process, a threshold value is also needed to determine whether a link should exist. Different selection of the threshold will significantly influence on the structure of reconstruction. These issues beg the question, how to provide a reasonable threshold. So far, there is no consensus on the best criterion of threshold, in particular for heterogeneous networks that are more complex due to two separate different thresholds for positive and negative links respectively. Furthermore, although numerous previous studies have been done in signed networks, research on projecting bipartite network to unipartite ones consisting of signed interactions is still rare. As it is still unclear how negative interactions impact on the function and application, more detailed and deep research should be done.

The aim of this work is to uncover heterogeneous interactions in online commercial networks. In that respect, we propose a novel approach to construct signed unipartite networks with heterogeneous interactions (i.e. positive or negative) between users from four online commercial website data. We also carry out the percolation analysis which studies the size of the largest component as a function of similarity thresholds. The percolation analysis reveals a phase transition phenomenon which is then used to determine the critical thresholds. To unveil different patterns of positive and negative interactions, the structural properties such as structure balance, motif and clustering of the giant components consisting of positive and negative links are investigated respectively. Finally, the roles of the negative links and weak ties are revealed by adding them back to the giant components.

## Results

Four real-world user-item bipartite networks, i.e., Movielens, Rate Your Music (RYM), Epinions and Douban, are used in this paper to explore heterogeneous interactions in online commercial networks. The raw data used in this paper include rating information with integer rating scales to one item by a user. This type of rating data are extracted from several major websites where users are allowed to express their opinion about certain online products such as movies, music and books. In the case of the 5-star ratings, a natural interpretation is to consider rating 3 as a neutral attitude and the ratings higher than it implies that the users truly like the product. This interpretation is widely used in a variety of research works studying online rating systems. In previous works which map the rating data to un-weighted bipartite networks^19,20^, only the high ratings are regarded as links. For instance, when investigating recommender systems, a common procedure is to completely neglect the ratings equal to or smaller than 3^21–23^. This is to avoid recommending such products to other users, as it is very likely that the users will dislike these products, leading to a low recommendation accuracy. However, this results in losing a large amount of information. In our study, in order to construct a signed network, We take into account all rating data. To be consistent with the literature, we regarded the ratings higher than 3 (in 5-star rating systems) and 5 (in 10-star rating systems) as likes and the rest ratings as dislikes in this paper. Specifically, we make used of the ratings smaller than 3 (in 5-star ratings) and 5 (in 10-star ratings) and model them as negative links (i.e. dislikes). That will be more accurate and significant to the recommendations, prediction and other applications based on this projection. Thus the raw rating data were modeled with bipartite networks consisting of user nodes and item nodes.

**Figure 1:**
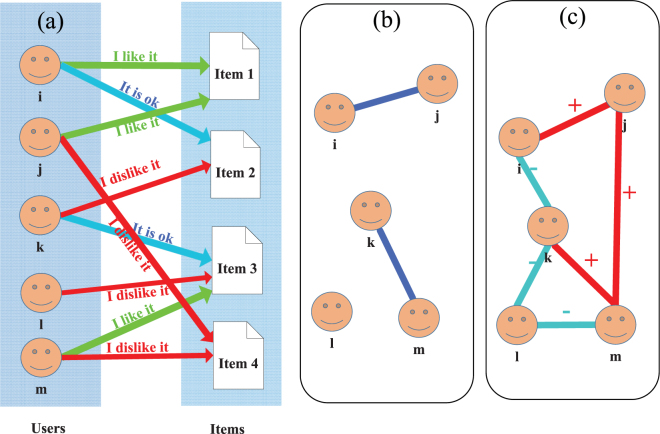
Illustration of projecting user-item bipartite network to unipartite user interaction network. (**a**) shows a typical user-item online rating network. The left column stands for users, the right ones for items. A link from a user to an item represents that the user has rated the item (red for dislike, green for like, blue for neutral, respectively). (**b**) presents the projected network through traditional Jaccard similarity without dislike ratings. The network is separated into three parts, with only homogenous links. (**c**) is the network projected by signed similarity. The network includes two different types of links (red is for positive, denoting similar preferences or opinions on the whole; blue for negative, dissimilar preferences, respectively).

The bipartite networks are further projected to the user side to construct a unipartite network in which the weight on a link between two users indeed represents the consistency of users’ attitude towards co-selected products. In order to obtain a unipartite network, similarity between two users is usually calculated in previous works, e.g. common neighbor^24^, Jaccard similarity^25,26^. The Jaccard similarity (see the detailed mathematical formula in the method section) is a way of comparing two users by looking at their fraction of common selected items (i.e. intersection of their items over the union of their items). The Jaccard similarity is calculated with only like links. To be more specific, we take the bipartite network in Fig. 1(a) as an example. In traditional studies, a rating of dislike will be removed (Thus the original red lines in Fig. 1(a) will be deleted). The obtained projected unipartite network by Jaccard similarity is shown as Fig. 1(b), which is separated into three connected components.

Being motivated by the Jaccard similarity, we propose a definition of signed similarity which can detect not only positive interactions but also negative ones between users. We modify the intersection of two users’ ratings sets in Jaccard as signed sum. We divide the ratings into two categories: like (i.e. the ratings higher than 3 for the 5-star rating cases and the ratings higher than 5 for the 10-star rating) and dislike (i.e. the ratings smaller than 3 for the 5-star rating cases and the ratings smaller than 5 for the 10-star rating). If two users’ opinions to one common item are the same, the numerator of signed similarity plus one. Otherwise, the numerator minus one. The numerator divided by the union size of the two users’ rating items is defined as signed similarity (See the method section for the detailed definition of the signed similarity). The signed similarity describes how much the two users agree with each other in items ratings. A positive value indicates that a product liked by a user is usually liked by the other user, which we define as a positive link (i.e. consistent taste or interest). On the contrary, a negative value indicates that a product liked by a user is often disliked by the other user. In this case, two users have opposite taste or interest, and we connect they by a negative link. Based on signed similarity, the user interaction network projected from the user-item bipartite network in Fig. 1(a) is shown in Fig. 1(c). With the negative links, the network becomes connected. Thus we construct a user-item bipartite networks with two type of links, i.e. likes and dislikes, from empirical data. With this bipartite networks, the signed similarity between users is computed for preparation.

### Correlation analysis between signed similarity and Jaccard similarity

**Figure 2:**
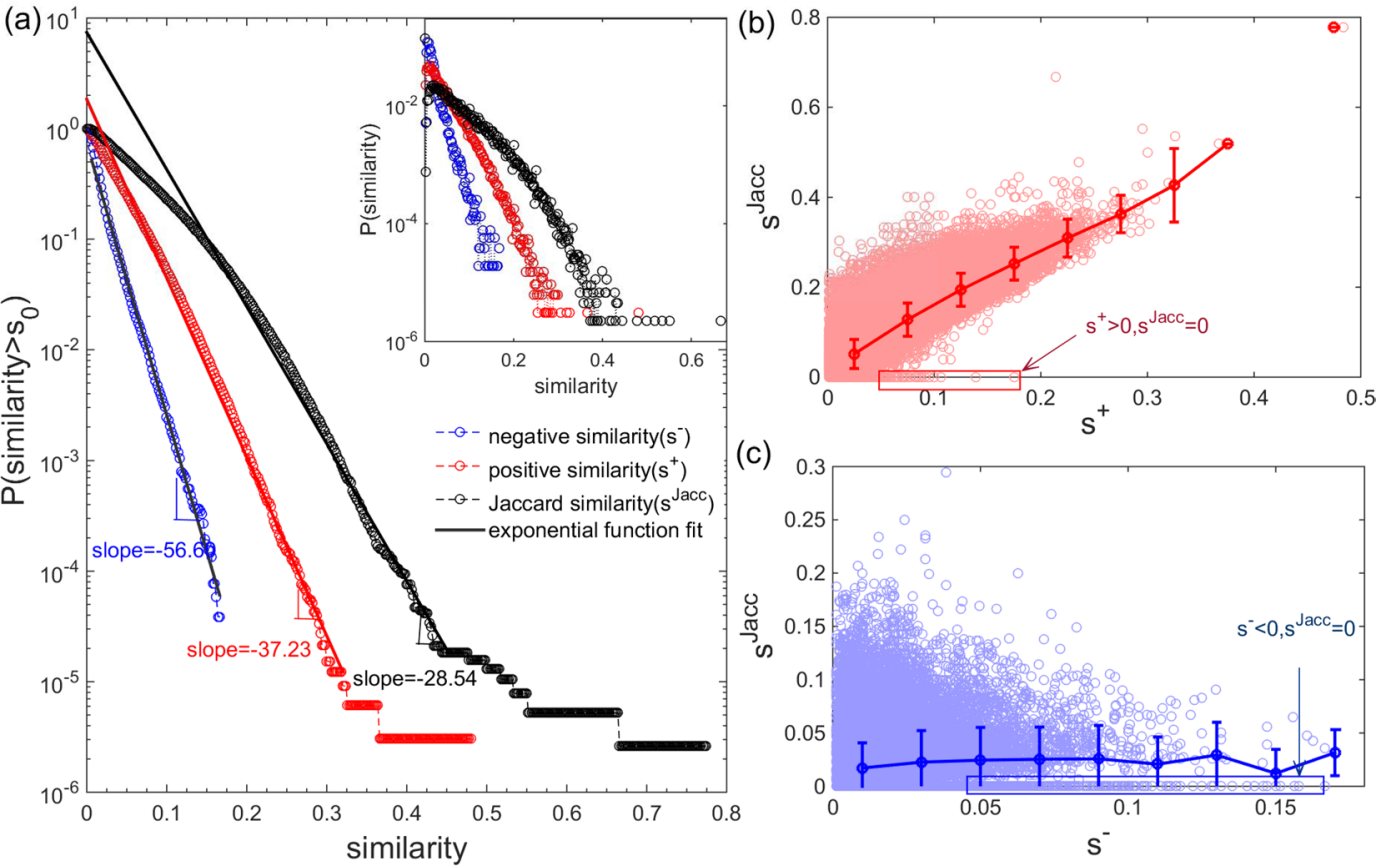
Relationship between signed similarity and Jaccard similarity (The results shown here are for the Movielens dataset). (**a**) The cumulative probability distributions of three similarities (i.e. Jaccard similarity (in black), positive similarity (in red) and negative similarity (in blue)) and probability density distribution in inset. They all roughly follow exponential distributions. The straight lines represent the least square fitting of the data points. (**b**,**c**) Present correlation between Jaccard similarity (*s* ^*Jacc*^) and positive similarity (*s* ^+^), negative similarity (*s* ^−^), respectively. The solid lines are average Jaccard similarity with respect to different signed similarity values (positive similarity (red line), negative similarity (blue line)) and error bar for the statistical standard deviation over different Jaccard similarity values. In general, Jaccard similarity increases along positive similarity increases, while almost remains unchanged when negative similarity rises. The scatter in the red and blue boxes indicate the cases that signed similarity is nonzero but Jaccard similarity is zero.

In the four projected unipartite networks, the proportion of negative links is significantly smaller than that of the positive ones. The distribution of the negative strength is narrower than that of the positive strength (see detail in SI Table. s1). We further turn to explore the relationship between signed similarity and Jaccard similarity. The distribution of signed similarity (*s* ^*sign*^, positive similarity (*s* ^+^) and negative similarity (*s* ^−^) separately) and Jaccard similarity (*s* ^*Jacc*^) (in terms of Movelens dataset) are displayed in Fig. 2(a). They all approximately follow exponential distributions but fitted with different parameters. The detailed relationship between these similarity measures can be seen in Fig. 2(b) and (c) in which scatter plots of Jaccard similarity as a function of positive similarity and negative similarity are presented. Jaccard similarity increases monotonically with positive similarity in Fig. 2(b), which implies a positive correlation between Jaccard similarity and positive similarity. As the same time, Jaccard similarity is always higher than positive similarity, which is partially due to the removal of dislike ratings. Noteworthy, there are certain cases where nonzero positive similarity cannot be detected by Jaccard similarity (identified in red box in Fig. 2(b)). On the contrary, average Jaccard similarity remains substantially constant while negative similarity varies as shown in Fig. 2(c). This suggests that Jaccard similarity is roughly unrelated to negative similarity. Besides, compared with the classic Jaccard similarity, the signed similarity can outperform it in recommendation accuracy when the known information is limited and effectively suppress mis-recommend dislike products for users (see the detail in SI (Supplementary Note 6)). Consequently, signed similarity is necessary to uncover the heterogeneous interactions and its role in detecting different types of relationship between users cannot be replaced by Jaccard similarity.

### Percolation analysis

To obtain a projected user-user unipartite network, the signed similarity between users has been calculated from the constructed user-item bipartite network with two type of links, i.e. likes and dislikes. But if all the users with non-zero signed similarity are connected, the resultant user-user social network will be very dense and no clear structural properties can be observed. A usual way is to use a threshold to filter out links with small similarity. Obviously, the resulted network’s topology strongly depends on the value of threshold. Thus a reliable and efficient threshold criteria is indispensable. However, so far there is no consensus on the best method of choosing threshold. It is notable that the variation of threshold describes a percolation process^27–30^. A representative percolation question is as follows: assuming a large two dimensional grid of edges, which are open or present with probability *p* (0 ≤ *p* ≥ 1) and closed or absent with probability 1 −*p* ^29,30^. Therefore, for a given *p*, what is the probability that an open path (meaning a path, each of whose links is an ‘open’ bond) exists from the top to the bottom? Percolation is typically studied in relation to the size of the largest open cluster (that is, the contiguous component of the graph of open edges). A classic example^29,30^ is the rapid emergence of a single giant component that encompasses a large fraction of nodes in random graph at a given probability of connection.

**Figure 3:**
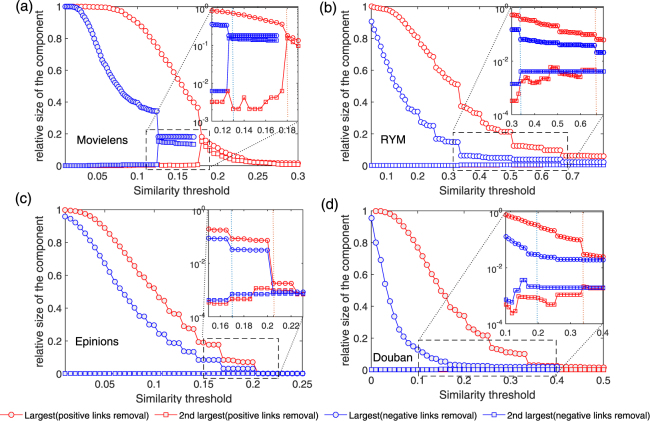
The first and second largest components’ sizes as function of similarity thresholds corresponding to four online user interaction networks. As we lower the positive and the absolute value of negative similarity threshold respectively, the size of the largest component increases in jumps when new modules emerge, grow and finally get absorbed by the largest component. The inset zooms in to the results around critical threshold. The largest component shows a jump while the second shows a peak, indicating a percolation transition at critical similarity threshold (*p*_*s*_ and *n*_*s*_).

In order to eliminate the weak links (i.e. links with small absolute value in the signed similarity), the percolation process is employed. Specifically, we set a certain threshold and remove all the links with the absolute value of the similarity smaller than this threshold. As the threshold gets larger, the resultant user-user social network becomes sparser. In our case, there are heterogeneous interactions with signed as positive or negative in the networks. Meanwhile, negative links are significantly fewer than positive ones and the two type links may play different roles in topological structure. Being aware of all above factors, two parameters are needed to express as the occupied probability *p*_*c*_ for positive links and *n*_*c*_ for negative links. In the paper, we investigate the dependence of the network giant component on the threshold, which is a standard percolation analysis procedure. We calculate the size of the largest and the second largest components as a function of positive threshold *p*_*c*_ and negative threshold *n*_*c*_. The results are shown in Fig. 3 (see also the heat map in Fig. s1 of SI). A large threshold value leads to isolated clusters, since only the strongest (i.e. more similar) users’ interactions are preserved. As threshold is lowered, the isolated clusters get progressively merged to larger entities and the emphasis is shifted toward large-scale properties of the spanning network. When decreasing the threshold from 1 to 0, we find obvious jumping phenomenon, i.e. the second largest component suddenly merges to the giant component when the threshold decreases with a very small value. This suggests percolation transition occurs. The inset in Fig. 3 presents the detailed behavior of jumps, that is, the second largest component size decreases sharply as the largest one increases at the critical point.

We have calculated a series of critical similarity at which a percolation transition takes place and giant component emerges. For efficiency purposes, we focus on the largest critical threshold values and under which the largest clusters are not extremely small. We call them strong similarity thresholds (*p*_*s*_ for positive threshold and *n*_*s*_ for negative one (listed in SI Table s2)). Then we link two users if their absolute signed similarity is larger than the corresponding threshold *p*_*s*_ or *n*_*s*_. In this way, the similarity threshold where this jumping occurs is recorded and the projected user-user social network is constructed with this so-called critical threshold.

### Topological structure features

**Figure 4:**
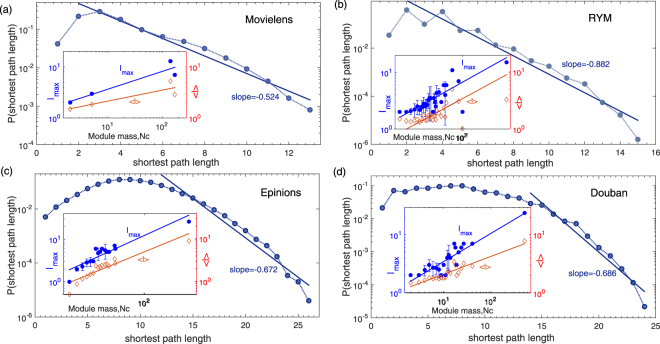
The probability density distribution of strong ties’ shortest path length in the largest modules. The tail of the distribution follows an exponential shape with straight line fitting in semi log scale. The insets show the functions of average shortest path length 〈*l*〉 (red diamonds) and maximum shortest path length *l*_*max*_ (blue dots) versus number of users (or module mass, *N*_*c*_). We use all modules appearing at signed threshold *p*_*s*_ and *n*_*s*_. Each point represents a bin average over all modules with the same size. The error bar is the standard deviation over the different modules with same size. Both 〈*l*〉 and *l*_*max*_ have a scaling relation with *N*_*c*_. The straight lines represent the least square fitting of the data points.

Next, we explore the properties of each projected unipartite networks extracted by percolation analysis. We investigate the length scales defined for each connected component: the average shortest path length 〈*l*〉, the maximum path length *l*_*max*_, and plot them as a function of the total number of nodes in each component (i.e. the scaling of each component, Module Mass, *N*_*c*_) in the insets of Fig. 4. The relationship shows power-law regime ${l}_{{\max }}\propto {N}^{{d}_{f}}$ for every network, for instance *d*_*f*_ = 0.347 in Movielens dataset (0.365 for RYM, 0.475 for Epinions and 0.487 for Douban). The exponent *d*_*f*_ quantifies how densely the users is covered by a specific module in topological structure, the parameters are computed with least-squares fitting. The scaling with 〈*l*〉 is consistent with the same equation, as seen in Fig. 4 insets.

We also study the structure of the largest giant components. Figure 4 shows the shortest path length distribution of each largest giant component. In all cases the results can be approximated with the exponential function especially in the range of tails. The fast decay of the exponential function indicates long distance links seldom occur between users under strong similarity restriction. Basic network measures of the giant components under investigation are presented in more detail in Table s3 of SI.

According to the structure balance theory^11^, the connected modules present excellent structure balance under the strong signed thresholds (*p*_*s*_, *n*_*s*_). In other words, the triangle motifs these networks are all structure balance triangles (that is, the product of three signs in a triangle motif is positive, see more explanation in Fig. s2 of SI). When we decrease the positive similarity threshold and fix the negative one at critical value as *n*_*s*_, positive links are added to strong connected component. As a result, the triangle motifs with signed links of positive, negative and positive are formed, which are the only type of non-balance structured triangle motif in this situation. The addition of positive links doesn’t change the structure balance much. When decreasing the absolute value of negative critical signed threshold and fixing positive one as *p*_*s*_, the negative links are added. In theory there are two types of non-balance structured triangle motifs (signed links of positive, positive and negative; or negative, negative and negative) could emerge. But in the empirical result only one type non-balance structured triangle motif (with links of all negative) emerges (see details for RYM dataset in Fig. s3 of SI). The level of structure balance reduces but still remains high with the proportion of balance triangle around 0.9 (the detail is plotted in Fig. s4 of SI).

**Figure 5:**
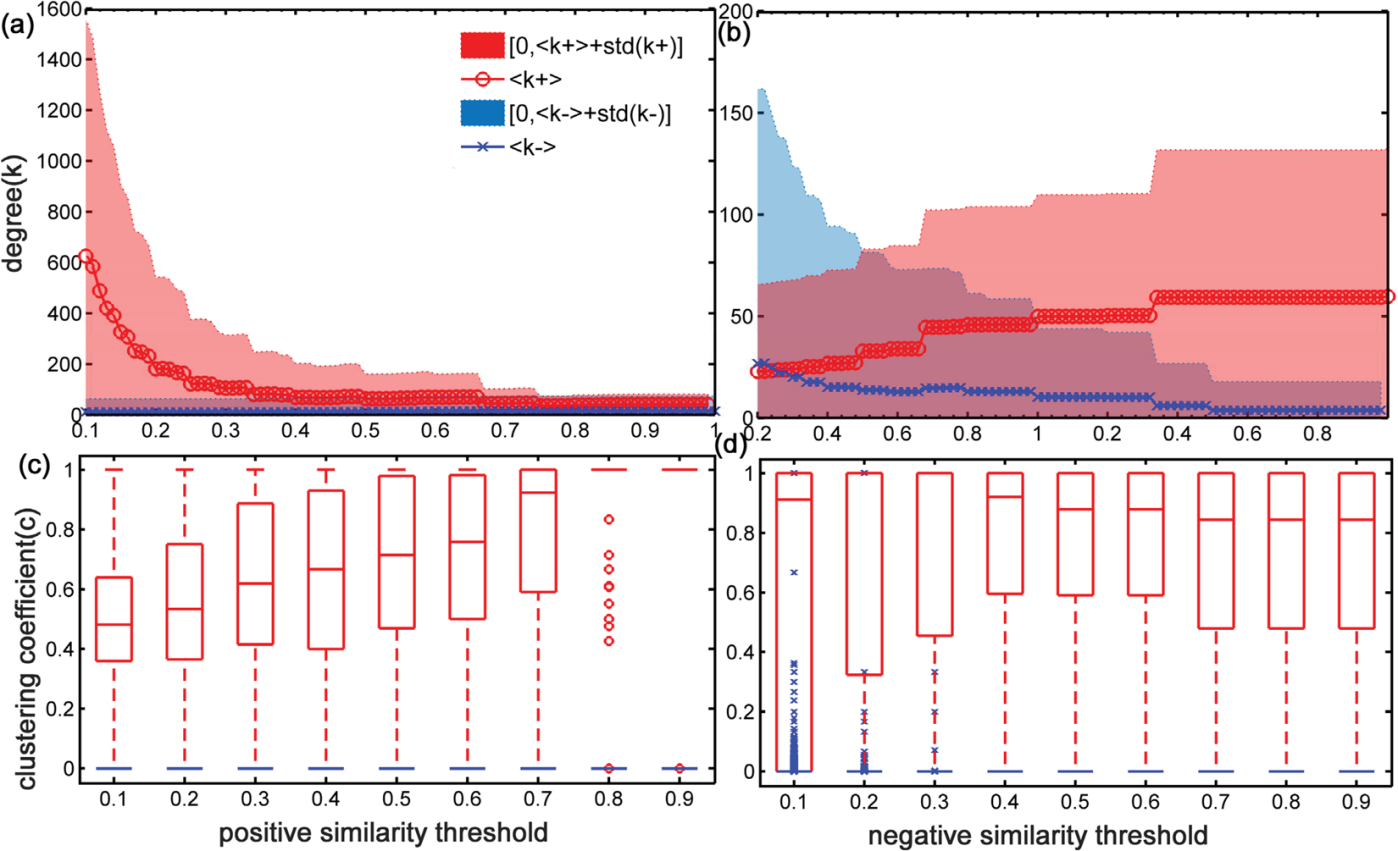
(For RYM dataset) Fixing one signed similarity threshold at strong critical point, the dependence of signed degree and signed clustering coefficient versus the other signed similarity threshold when the positive and negative links are respectively removed from the network (Red represents positive network statistics and blue represents negative ones). Top Panel: plot of signed degree vs. signed similarity threshold. The solid line corresponds to average signed degree over all nodes in the largest component (red circle for average positive degree, 〈*k*+〉; blue cross for average negative degree, 〈*k*−〉). The shaded area indicates standard deviation interval(red area for (0,〈*k*+〉 + *std*(*k* + )], blue area for [〈*k* −〉 + *std*(*k* −),0)). (**a**) With negative similarity threshold fixing at *n*_*s*_, the dependence of signed degree vs. positive similarity threshold when the positive links are removed from the network. (**b**) With positive similarity threshold fixing at *p*_*s*_, the dependence of signed degree vs. negative similarity threshold when the negative link are removed from the network. The average positive degree and its standard deviation decrease rapidly as the positive similarity threshold increases (the negative similarity threshold remains critical threshold), while the negative one does not change much. When the positive threshold is fixed and the absolute value of negative one is heightened, the negative degree and its interval lower, with positive one increases slightly. Bottom Panel: boxplot of signed clustering coefficient vs. signed similarity threshold. (**c**) With negative similarity threshold fixing at *n*_*s*_, the dependence of signed clustering coefficient vs. positive similarity threshold when the positive links are removed from the network. (**d**) With positive similarity threshold fixing at *p*_*s*_, the dependence of signed clustering coefficient vs. negative similarity threshold when the negative links are removed from the network. The figures indicate the positive one is significantly higher than the negative one at all similarity threshold.

We proceed further by investigating the structure features of the largest components with a particular focus on network statistics variation as changing signed threshold respectively. When absolute threshold increases, the number of signed links in the largest component reduces almost exponentially with several sharp drops (see SI Fig. s5). Along with the change of signed similarity thresholds, the signed degree and signed clustering coefficient of nodes in the largest components vary, as presented in Fig. 5 (for RYM dataset as an example). Bottom panel of Fig. 5((c),(d)) is the boxplot of signed clustering coefficient (*c*), in which the positive clustering coefficient is always significantly higher than the negative one. This property is robust as it does not depend on signed threshold parameter. This suggests users interact more closely through positive links than negative ones. In terms of degree, as shown in Fig. 5(a) when we raise the positive similarity threshold with fixing negative one at critical point (*n*_*s*_), average positive degree (〈*k *+〉) in the largest component decreases extremely fast accompanied by some jumps, as the same time the standard deviation (*std*(*k* + )) reduces in a similar way. On the other hand, average negative degree (〈*k* −〉) and its standard deviation (*std*(*k* −)) changes slightly. On the contrary, when the absolute value of negative similarity threshold is raised with positive one fixed as *p*_*s*_, average negative degree (〈*k* −〉) in the largest component and its standard deviation (*std*(*k* −)) both lower substantially as expected. However, average positive degree (〈*k* + 〉) only increases slightly (see in Fig. 5(b)). This phenomenon can be explained by the different roles that the positive and negative links play in the social networks. It could be the case that negative edges trend to link inter communities while positive edges prefer intra community. When negative links are deleted, more links between communities are cut so that the sparse affiliated communities are more likely removed, which makes the density of positive links increase in the largest component.

### Functions of the negative links and weak ties

**Figure 6:**
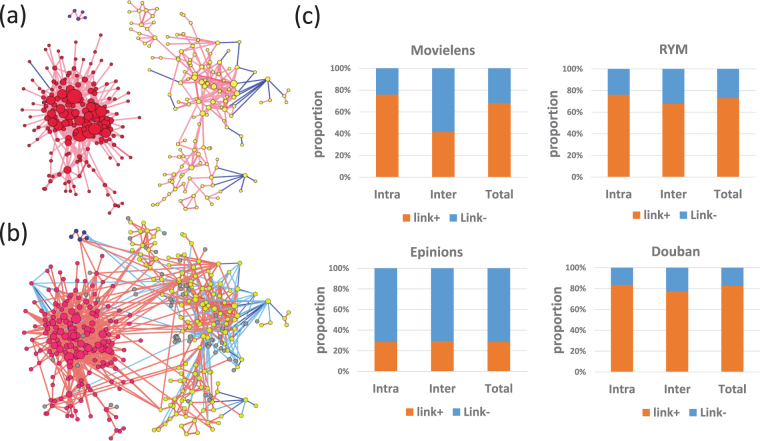
Functions of negative links and weak ties. (**a**) Three separate modules are identified at *p*_*s*_ = 0.18, *n*_*s*_ = −0.13 in dataset Movielens, the colors of nodes correspond to different connected components. The red lines stand for positive interactions, and blue ones for negative ones. (**b**) When the thresholds are lowered to *p*_*w*_ = 0.175 and *n*_*w*_ = −0.1, the isolate components turn to be connected by weak ties. The grey dots are the nodes added from (**a**) when the thresholds are lowered. (**c**) 100% stacked column graph of distribution of new added weak signed links for inter or intra separate modules. Colors present different signed links, (i.e. red for positive links (link +), blue for negative links (links −)). The first column (Intra) presents the weak signed links distribution within original components, the second column (Inter) is for the proportion between original components, the last column is fraction of signed links in total new added weak links. The proportion of negative weak links between components is higher than within isolate components in all cases, which indicates the preference of weak negative links in connecting separate modules.

To verify the hypothesis of different role for signed links mentioned above, we lower the absolute value of signed similarity thresholds to include weaker ties (new added links), hence the original separated modules will be connected through added shortcuts. A typical scenario in MovieLens data is depicted in Fig. 6. Three isolate largest components are identified in Fig. 6(a) at strong critical similarity threshold *p*_*s*_ = 0.18, *n*_*s*_ = −0.13. When we lower the thresholds’ absolute value to *p*_*w*_ = 0.175 and *n*_*w*_ = −0.1, the modules merge to a connected network (Fig. 6(b)). Furthermore, we compute the original shortest path length between nodes connected with weaker ties. The probability distribution reveals a power-law behavior (as shown in SI Fig. s6) which suggests the optimal wiring^31^. Figure 6(c) is 100% stacked column graph of new added weak signed links’ distribution (positive links (link+) and weak negative links (link−)) between(inter) or within(intra) separate modules. The proportion of negative weak ties inter-modules is significantly more than intra-modules. The observation of such a distribution of weaker signed links suggests that the links with different signs may play different roles in users’ interaction networks. Users are more likely gathered through positive links (similar opinions), and the separate modules are mainly collapsed with weaker negative links as shortcuts.

## Discussion

Online commercial systems provide a large amount of users’ purchase and rating data for understanding human behaviors. Uncovering user interaction structure and purchase preference patterns are crucial for a wide range of applications, such as recommending relevant information for users and providing personalized service. This type of systems are usually modeled by bipartite networks. The traditional methods for understanding such bipartite networks usually project them to unipartite ones. Considering negative ratings are also meaningful for users’ purchase choice, such online commercial networks should be analyzed with the approaches that account for both types of interactions. In this paper, we decipher signed interactions (positive or negative) in online commercial networks based on both like and dislike ratings. The resultant unipartite networks preserve much more information of bipartite user-item networks.

In spite of the vast and growing literature on signed networks, the detection and function of negative links and weak ties remain a largely unexplored problem. So far there is no consensus on the definition of the negative links and criterion for threshold to construct the links. We give a novel method to detect and uncover the heterogeneous interaction between users, then percolation analysis is employed to determine the similarity’s threshold, which reveals a phase transition phenomenon. We implement a complex network analysis to understand heterogeneous interaction between users, and study the role of negative links and weak ties in users’ interaction networks. The statistical features and the scaling law of the giant component consisting of positive and negative links are investigated respectively. The heterogeneous user interaction networks present strong structure balance overall, indicating that users are more likely clustered through positive links rather than negative ones. The different location of signed links in weak ties implies different function of the negative links compared to positive ones.

Surely there is no direct interaction between users in the user-user social network we constructed. However, it actually reflects a type of indirect relation between users. The approach of mining indirect relations in real systems is widely adopted in related research. For instance, the product space, a virtual network between exported products, is projected from the country-product international trading network with a proximity metric^32,33^. By constructing this network, the development of countries is revealed to be a diffusion process. In addition, the disease network, a virtual network describing the symptom similarities between diseases, is constructed by projecting the disease-symptom bipartite network to the disease side^34^. This network provides important reference for disease etiology research and drug design. Similarly, in our work a virtual user-user social network is constructed from the original user-item bipartite rating networks, leading to a deeper understanding of the complex organization of users’ interests. The present results provide a unique view to uncover heterogeneous interactions in online commercial networks. This representation of signed interactions has offered new insight in many processes across different disciplines. The suggested phase transition phenomenon features as well as the negative links and weak ties patterns can be applied to other online social networks and other similar bipartite networks in future research. Finally, this research provides a basis for the study of filtering irrelevant information, link prediction and personal recommendation.

## Methods

### Data Description

Four empirical datasets, i.e., the MovieLens, the RYM, the Epinions and the Douban, are used to decipher the heterogeneous interactions in online commercial networks. The MovieLens data^35^ is available at http://www.grouplens.org/. It contains 1682 movies and 943 users who rated the movies using the integer scale from 1 (worst) to 5 (best). The original data contains 10^5^ ratings. The RYM data^36^ is publicly available on the music ratings website RateYourMusic.com. The ratings in RYM are given on the integer scale from 1 to 10 (i.e., worst to best). The data consists of 24,775 users, 5267 albums, and 667,437 links. The Epinions (http://www.epinions.com, an online product rating site) data is download from KONECT (http://konect.uni-koblenz.de/networks/epinions-rating)^1^ and a smaller data set is extracted by randomly sampling of the whole records of user activities. It finally consists of 28,487 users who rated a total of 138,675 different items, and 649,210 links. Douban data^37^ is obtained from http://www.douban.com/, which is a Chinese social network site allowing registered users to record information and create content related to films, books, music and so on. Here, we use the data with 1,384,187 ratings by 20,677 users on 185,467 music. The last two data are both five-level rating systems same as MovieLens.

### Signed Similarity

To identify heterogeneous interactions between users from the original bipartite online commercial networks, we propose the signed similarity metric (*s* ^*sign*^) which works similarly to Jaccard similarity.

For two given users *i* and *j*, the Jaccard similarity is defined as ${s}^{Jacc}ij=\frac{{\rm{|}}{\rm{\Gamma }}(i)\cap {\rm{\Gamma }}(j){\rm{|}}}{{\rm{|}}{\rm{\Gamma }}(i)\cup {\rm{\Gamma }}(j){\rm{|}}}$, where Γ(*i*) is the set of items that user *i* gives like ratings only. The cardinality of one set *A* denoted |*A*| counts how many elements are in the set. For user pair (*i*, *j*), the set of common items is denoted as Γ(*i*) ∩ Γ(*j*), the intersection between two sets. The union between Γ(*i*) and Γ(*j*) is denoted as Γ(*i*) ∪ Γ(*j*) and reveals all items which are in either set.

Inspired from the literature^38,39^, the definition of the heterogeneity is proposed as signed similarity. Mathematically, the formula for signed similarity(*s* ^*sign*^) reads

1 $${s}_{ij}^{sign}=\frac{\sum _{\alpha \in {\rm{\Gamma }}(i)\cap {\rm{\Gamma }}(j)}sign(rating(\alpha ,i),rating(\alpha ,j))}{|{\rm{\Gamma }}(i)\cup {\rm{\Gamma }}(j)|},$$

where Γ(*i*) is the set of items that user *i* has reviewed not only like ratings but also dislike ratings. *rating*(*α*, *i*) stands for the rating to item *α* by user *i*, which is categorized as like and dislike. *sign*(*rating*(*a, i*), $$rating(\alpha ,j))=(\begin{array}{cc}1, & \,if\,rating(\alpha ,i)=rating(\alpha ,j)\\ -1, & \,if\,rating(\alpha ,i)\ne rating(\alpha ,j)\end{array}$$, that means, if user *i* and user *j* have same attitude to item *α* (i.e. both like rating or both dislike rating), positive one should be added to numerator, otherwise, numerator will minus one. Signed similarity measures how similar one user is to another in the context of rating items (such as movies and music). If the products liked by a user is usually liked by the other user, we connect them with a positive link (i.e. consistent taste or interest). On the contrary, if the products liked by a user is often disliked by the other user, it is natural to connect them by a negative link (opposite taste or interest). In other words, a positive similarity indicates that the interests of two users are consistent, while a negative similarity indicates that the interests of two users are opposite.

## Electronic supplementary material

Supplementary information
