# Supplementary material for: Uncovering heterogeneous interactions in online commercial networks
Source: Sci Rep. 2017 Dec 8;7:17209. doi: 10.1038/s41598-017-17410-1 (PMC5722876; doi:10.1038/s41598-017-17410-1)
Supplement: Supplementary file 1 — Supplementary information [file 41598_2017_17410_MOESM1_ESM.pdf]

Supplementary Information

Uncovering heterogeneous interactions in online commercial networks

Fangfeng Zhang, An Zeng, Bowen Ma, Ying Fan and Zengru Di

Supplementary Note 1: Data description.

Four empirical datasets, i.e., the MovieLens, the RYM, the Epinions and the Douban, are used in this paper. To decipher the heterogeneous interactions, signed similarities are calculus between users who rate at least one common item. Thus the online bipartite networks are projected to unipartite user interaction networks. In the unipartite user interaction networks, nodes represent users, the links between two nodes means non-zero signed similarity. The links (interactions) between users are either positive or negative. The basic description of user interaction networks is shown as Table S1. The basic description includes: the number of users (N), the total number of links (E), the number of positive links ($link+$) and its proportion in the total links, the number of negative links ($link-$) and its proportion, and interval of positive similarity (s^+) and negative similarity (s^-). In this paper, the MovieLens data contains 943 users, the other three empirical datasets all contain more than twenty thousands users. In all empirical unipartite user interaction networks, the positive links are much more than negative ones. For instance, the MovieLens user interaction network contains total 379,456 links, and 86.39% of which are positive links.

Supplementary Note 2: Percolation analysis.

To determine a proper signed threshold for cutting the links in reconstruction process, improved percolation analysis with two parameters (p_c as occupied probability for positive links, n_c for negative ones) is carried out for every user interaction network. We calculate the size of the largest components as a function of positive threshold p_c and negative threshold n_c. The result of heat map is shown in Fig. S1. The color denote the proportion of the number of nodes in the largest component to the total nodes in original network. The color dose not change continuously which implies percolation transitions occur.

At every percolation transition point, we get one critical signed similarity threshold. Thus we calculus a series critical signed similarity threshold. In this paper, we focus our attention

on the largest critical threshold values and under which the largest clusters are not extremely small (which are called as strong critical similarity threshold p_s or n_s shown in Table S2).

Supplementary Note 3: Topological property of strong user interaction modules.

Now we focus on the topological structural property of the strong user interaction network under the strong critical signed similarity thresholds (p_s, n_s) (presented in Table S3). The topological structural properties include network size (number of users, nodes, N); links: total number of links (E), number of negative links ($link-$) and the proportion of positive links in all links; triangle motif: total number of triangles (in this case, all the triangle motifs contain structure balance, that is , the product of all links in the triangle motif is positive), the number of triangle motif with links of positive, negative and negative (Tri_p_nn); signed degrees: the average positive degree ($\langle k+\rangle$) with its standard deviation ($std(k+)$), the average negative degree ($\langle k-\rangle$) with its standard deviation ($std(k-)$), the Pearson correlation coefficient ($Corr$) between positive degree and negative degree; signed clustering coefficient(signed c): the average positive clustering coefficient ($\langle c+\rangle$) with its standard deviation ($std(c+)$), the average negative clustering coefficient ($\langle c-\rangle$) with its s-tandard deviation ($std(c-)$); shortest path length(L): the maximum path length l_{max}, the average shortest path length $\langle l\rangle$ and its standard deviation($std(l)$).

Supplementary Note 4: Structure balance analysis.

Structural balance was first proposed by sociologists Heider [1]. According to this theory, the triangle motifs are divided to structure balance triangles and non structure balance triangles. One triangle motif of structure balance means the product of all links in the triangle motif is positive (see explanation in Fig. S2). In this paper, all user interaction networks under strong signed critical threshold (p_s, n_s) are structure balance.

When we fix one signed similarity threshold at the strong critical threshold and change another similarity threshold, the number of triangle motif varies as shown in Fig. S3. The negative links addition reduces the structure balance level of whole network by adding the triangle motifs with three negative links. The positive links addition leads emergence of the triangle motifs with two positive and one negative links. Fixing one signed similarity

threshold at strong critical point, one kind of signed links adding can only add only one type non-structure balance triangle motif into the original strong heterogenous networks in empirical study.

The structure balance level of a whole network is indicated by the fraction of structure balance triangles in all triangle motifs. But in empirical signed networks, positive links are significantly more than negative links. So the triangles with negative links are naturally much less than triangles with all positive links. The index of fraction of structure balance triangles in all triangle motifs will not be significant with signed links removal. Thus, we modify the index to fraction of triangles with one positive link and two negative links in all triangles except triangles (in Fig. S4). In the empirical result, the positive links removal dose not change much the structure balance level. The more negative links in the networks, the lower level of structure balance of the networks. As long as the negative links removal, the whole networks reach strong structure balance soon.

Supplementary Note 5: Weak ties distribution

When we fix one similarity threshold at strong critical value and raise the other threshold's absolute value, the corresponding signed links are removed from the networks. The number of signed links decreases with some jumps when the signed similarity threshold increases. The number of signed links depending on the corresponding threshold's changing are shown in Fig. S5.

When we lower the absolute signed threshold below the strong critical similarity threshold, weaker ties are incorporated to the network. We compute the original shortest path length between nodes connected with weaker ties. The probability distribution reveals a power law behavior (as shown in Fig. S6) which implies the optimal wiring [2].

Supplementary Note 6: Recommendation performance.

We compare the recommendation performance of the signed similarity and Jaccard similarity with the user-based collaborative filtering recommendation algorithm. Specifically, we divide the raw real data into two parts, i.e. a training set E^T containing $1-q$ fraction of the data and a probe set E^P consisting of the rest q data. To generate recommendation results,

the signed similarity and Jaccard similarity are calculated respectively in the training set which contains both positive and negative links. The Jaccard similarity is calculated with only positive links while signed similarity is computed with both types of links. The probe set is used to evaluate the recommendation performance. It contains rating information of users, only the products connected by partial ratings are considered as the ones liked by the users for, i.e. the ratings higher than 3 for the 5-star rating cases and the ratings higher than 5 for the 10-star rating. We evaluate the recommendation results via four metrics including ranking score, precision, personalization and novelty [3]. The results are presented in Fig. S7 (for movielens data) and Fig. S8 (for epinions data). One can see that the recommendation accuracy by signed similarity is better than that of Jaccard similarity especially when q is large (i.e. the training set is sparse). In this case, the positive links are too few to accurately estimate the similarity between users and it is important to extract additional information from negative links. Moreover, the signed similarity can in general outperform the Jaccard similarity in recommendation diversity. Finally, we study the disliked products in the recommendation lists generated by these two similarity measures, as shown in Fig. S9. One can see that not only the signed similarity can suppress the ranking of these dislike products in the recommendation lists (as measured by ranking score), but also the top-20 recommendation lists include much fewer dislike items (as measured by precision). In summary, compared with the classic Jaccard similarity, the signed similarity can outperform it in recommendation accuracy when the known information is limited and effectively suppress mis-recommend dislike products for users.

Supplementary Figures

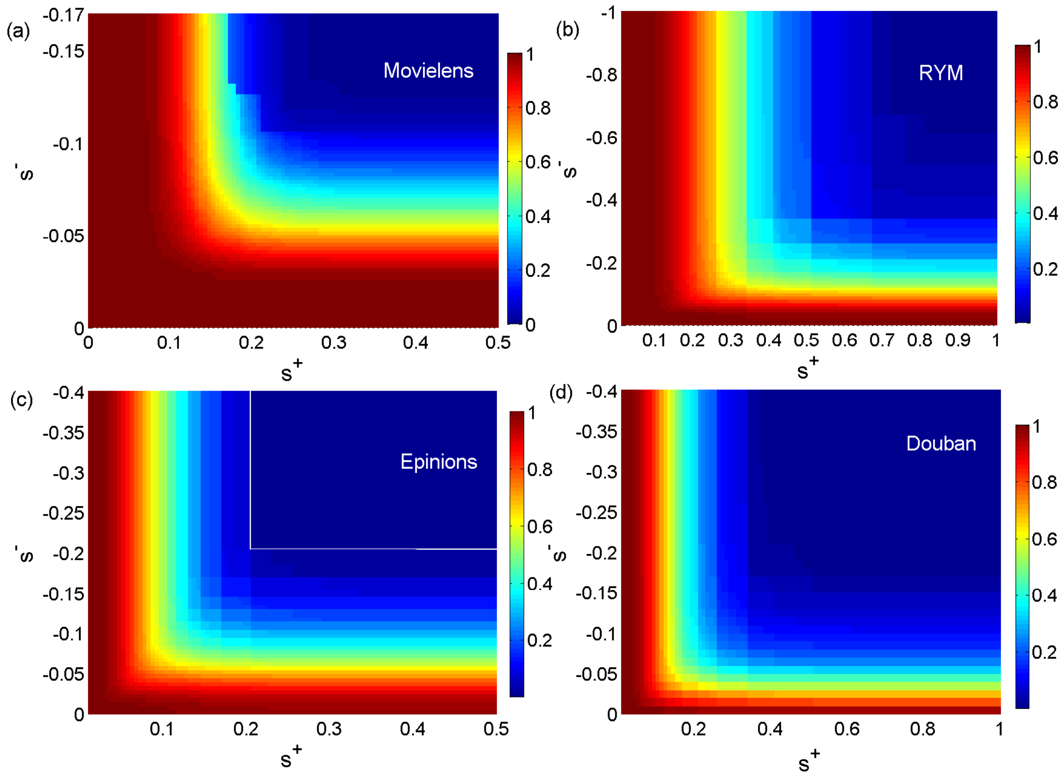

Supplementary Fig. S 1: (Color online) Heat map of the largest component proportion versus the signed similarity threshold (positive similarity(s^+), negative similarity(s^-)) through percolation analysis. The color presents the proportion of the largest component in the total number of nodes. The change of color is not continuously when the signed similarity threshold varies. That implies percolation transitions.

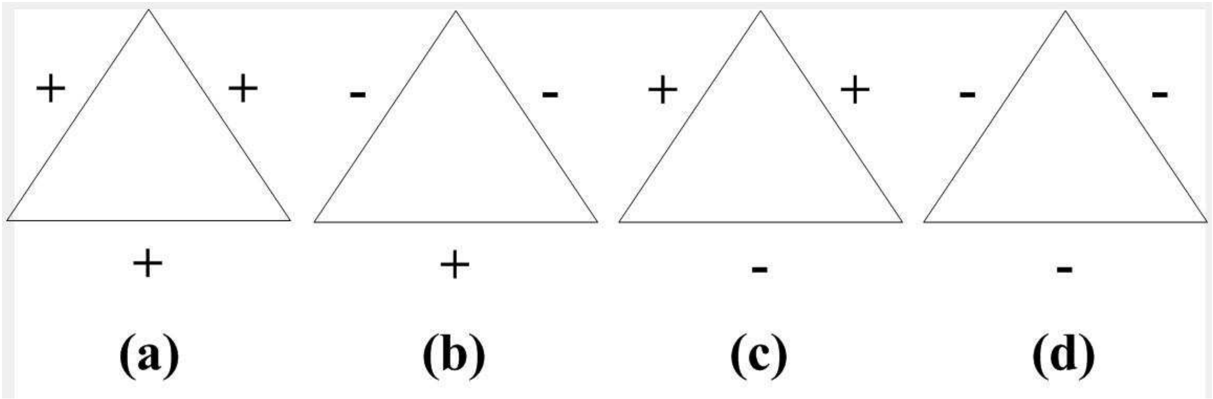

Supplementary Fig. S 2: (Color online) Illustration of structure balance theory in signed network. In signed network there are four types of triangle motif. According to structure balance theory, if the product of all links in a triangle motif is positive, the triangle motif is called strong structure balance triangle. That is, (a)(b) are structure balance triangles, (c)(d) are not structure balance triangles.

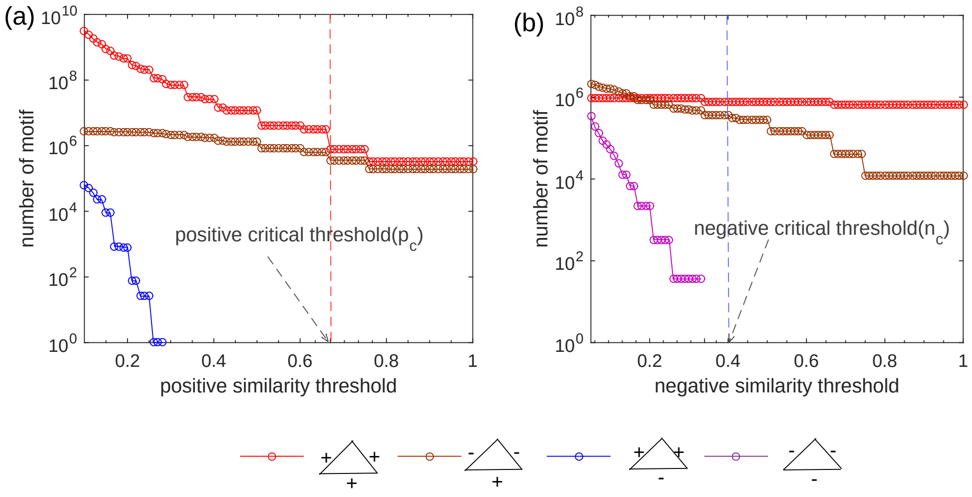

Supplementary Fig. S 3: (Color online) The number of different type motif versus one signed similarity threshold when the other is fixed at strong critical threshold (for RYM dataset). (a) Fixing negative similarity threshold at n_s, the number of triangle motif vs. positive similarity threshold. The number of triangles with three positive links decreases along with several jumps when the positive similarity threshold increases. The number of triangles with links of negative, negative and positive decrease slightly. Only one type of non-structure balance triangle (triangle with positive, positive and negative links) exists in the situation, and its number reduce extremely fast. (b) Fixing positive similarity threshold at p_s, the number of triangle motif vs. negative similarity threshold. The number of the other structure balance triangles with links of negative, negative and positive decreases along with several jumps when the absolute value of negative similarity threshold increases. The number of the other structure balance triangles with three positive links decrease slightly. Only one type of non-structure balance triangle (triangles with three negative links) exists in this situation, and its number reduce extremely fast.

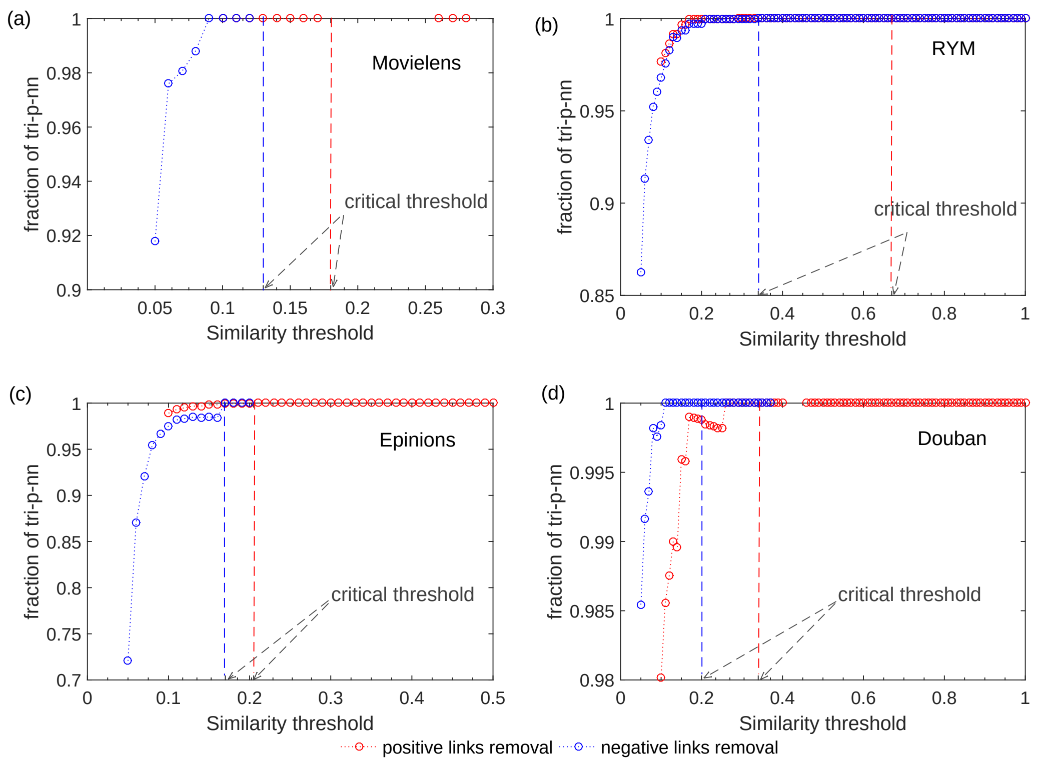

Supplementary Fig. S 4: (Color online) Fraction of triangles with one positive link and two negative links($tri{-}p{-}nn$) in all triangles except triangles with three positive links when the positive and negative links are removed from the network respectively. As a whole the structure balance level of empirical user interaction networks remains high when one signed similarity threshold fixes at strong critical threshold. The red dashed line with red circle presents the fraction with the positive links removal when the positive similarity threshold is rising and the negative similarity fixes at critical threshold (n_s). The fraction raise extremely fast at the very beginning and the fraction reaches 1 quickly. But even the minimal fraction is still around 0.95, the positive links removal dose not change much the structure balance level. The blue dashed line with blue circle presents the fraction with the negative links removal when the absolute value of negative similarity threshold is rising and the positive similarity fixes at critical threshold (p_s). The more negative links in the networks, the lower level of structure balance of the networks. The fraction also reaches 1 quickly and maintains 1 after critical threshold.

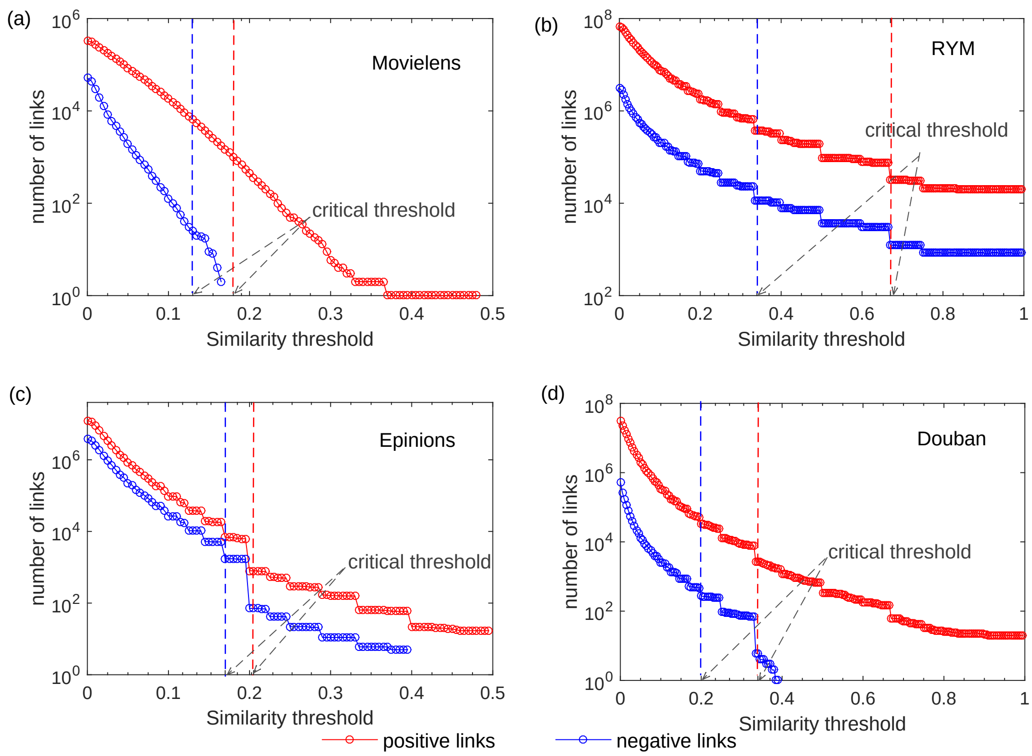

Supplementary Fig. S 5: (Color online) The number of signed links in the largest component versus the absolute value of signed similarity threshold. When one signed similarity threshold is fixed at strong critical value, the corresponding signed links are removed from the networks as the absolute value of the other similarity threshold increasing. The red line means the positive links changes as the positive similarity threshold varies, the blue for the negative ones. The number of signed links in largest components reduces almost in an exponential form with several sharp drops.

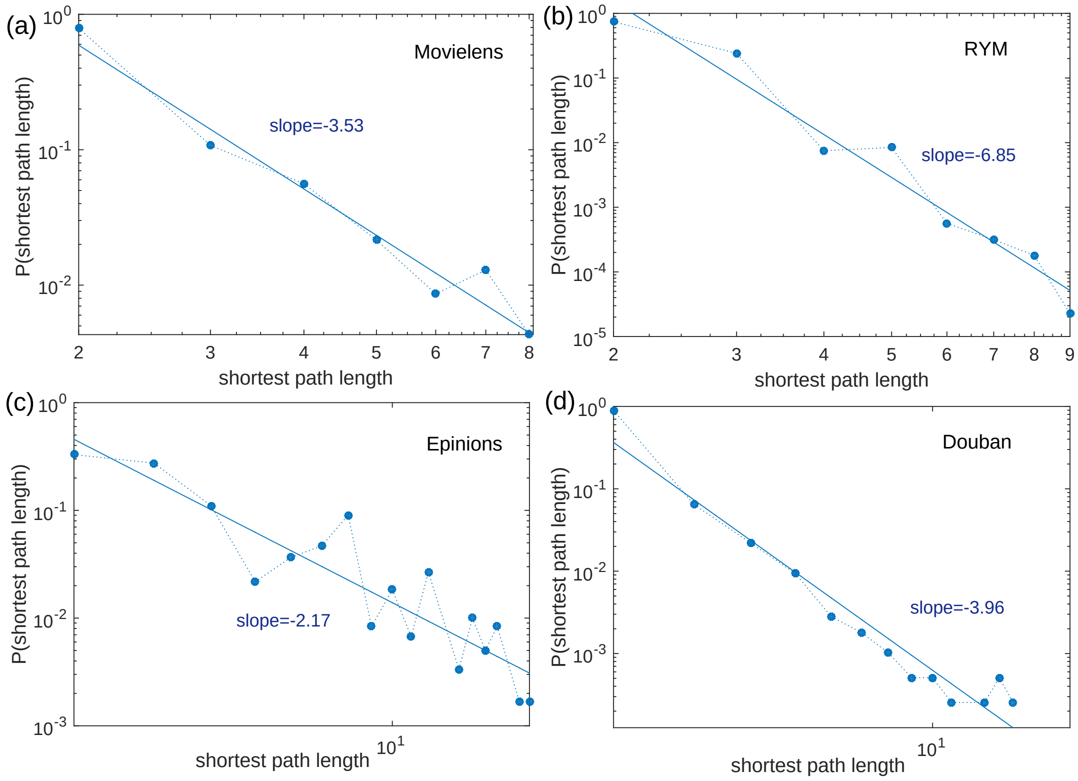

Supplementary Fig. S 6: (Color online) The probability density distribution of original shortest path length between nodes connected with weak ties. The probability distribution reveals a power law behavior.

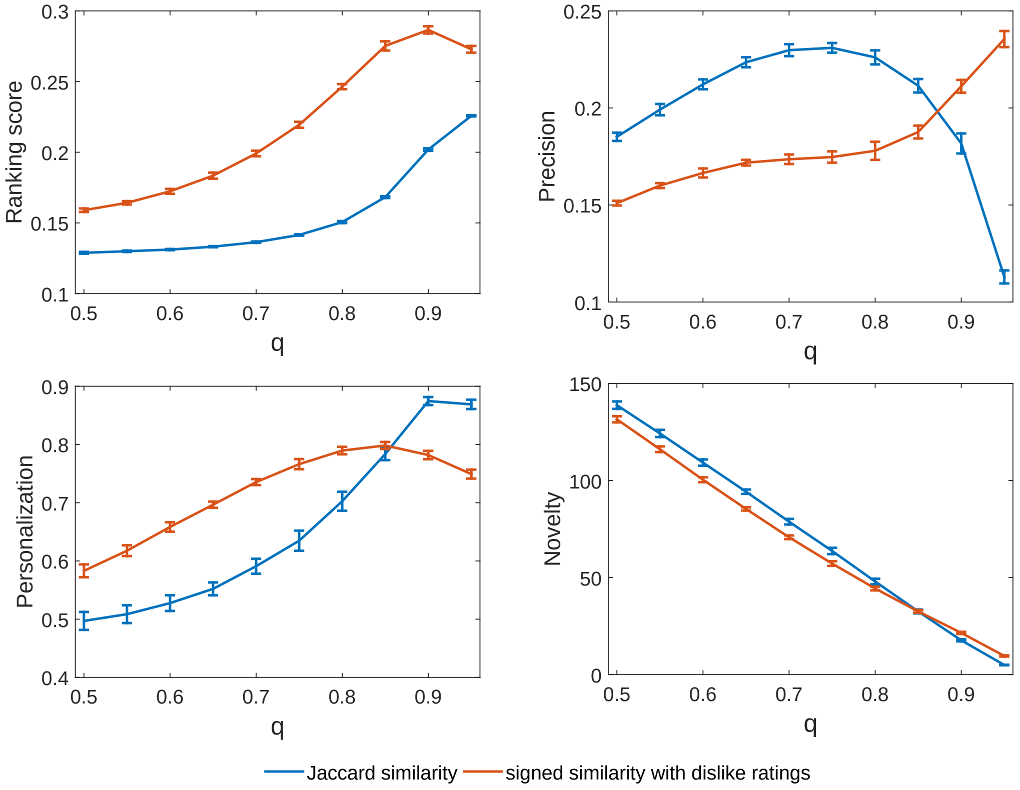

Supplementary Fig. S 7: (Color online) Signed similarity's and Jaccard similarity's ranking score, precision, personalization and novelty for Movielens data. The orange curve represents the performance of the signed similarity, the blue one for Jaccard similarity. The error bar indicates the standard deviation value obtained by ten independent training set-probe set divisions.

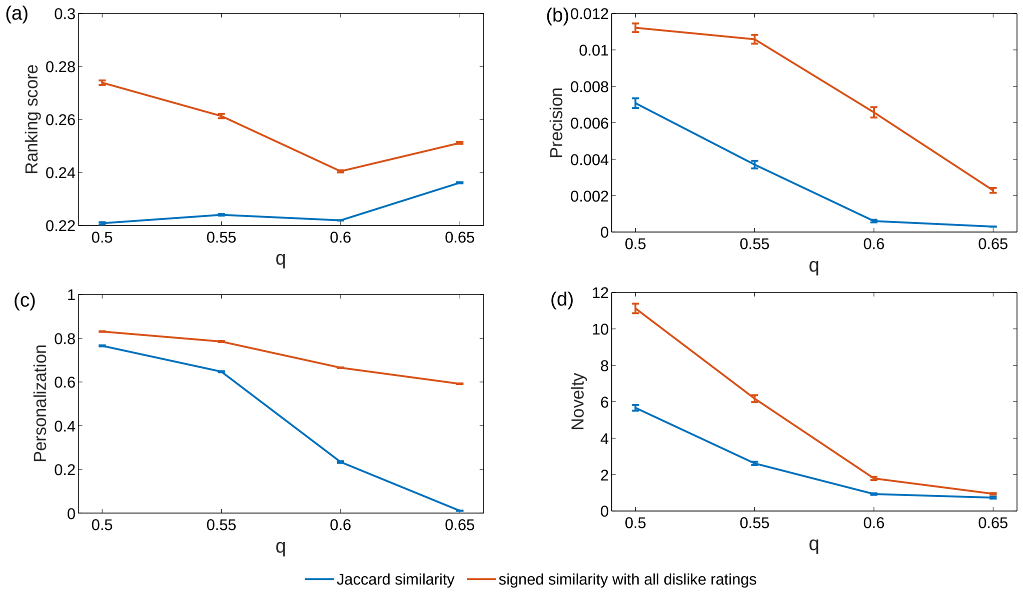

Supplementary Fig. S 8: (Color online) Signed similarity's and Jaccard similarity's ranking score, precision, personalization and novelty for Epinions data. The orange curve represents the performance of the signed similarity, the blue one for Jaccard similarity. The error bar indicates the standard deviation value obtained by ten independent training set-probe set divisions.

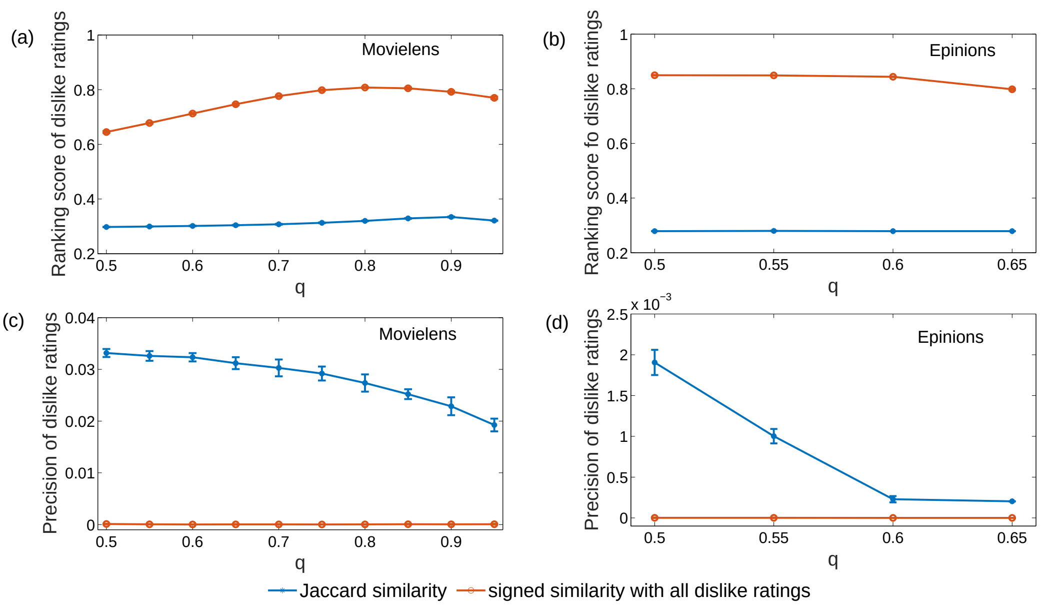

Supplementary Fig. S 9: (Color online) Signed similarity's and Jaccard similarity's ranking score, precision to dislike ratings (for Movielens and Epinions data). The orange curve represents the performance of the signed similarity, the blue one for Jaccard similarity. The error bar indicates the standard deviation value obtained by ten independent training set-probe set divisions.

Supplementary Table

Supplementary Table S I: The description of projected user interaction networks in empirical data. Structural properties include network size (number of users, nodes, N), number of links (E), number of positive links ($link+$) and the proportion of positive links in all links, number of negative links ($link-$) and the proportion, the interval of positive similarity (s^+) and the interval of negative similarity (s^-).

	Nodes (N)	Links (E)	Positive links ($link+$)	Negative links ($link-$)	Positive sim (s^+)	Negative sim (s^-)
**Movielens**	943	379,456	327,687 (86.39%)	51,769 (13.61%)	(0, 0.4839]	[-0.1667, 0)
**RYM**	$24,775$	$70,842,622$	67,727,932 (95.6%)	3,114,690 (4.4%)	(0, 1]	[-1, 0)
**Epinions**	$28,422$	$16,309,761$	12,555,677 (76.98%)	3,754,084 (23.02%)	(0, 0.5]	[-0.4, 0)
**Douban**	$20,677$	$32,271,899$	31,732,778 (98.33%)	539,121 (1.67%)	(0, 1]	[-0.3939, 0)

Supplementary Table S II: **The strong critical signed similarity thresholds through percolation analysis.**

	**positive threshold** (p_s)	**negative threshold** (n_s)
**Movielens**	0.18	-0.13
**RYM**	0.670	-0.34
**Epinions**	0.205	-0.17
**Douban**	0.34	-0.2

Supplementary Table S III: The topological structural properties of large modules in empirical data under strong critical signed similarity threshold.

		**Links**		**Triangle motif**		**Signed degree**			**Signed c**		**L**	
	Nodes N	Total E	$link-$ (proportion)	Total	Tri_p_nn (proportion)	$\langle k+\rangle$ ($std(k+)$)	$\langle k-\rangle$ ($std(k-)$)	$Corr$	$\langle c+\rangle$ ($std(c+)$)	$\langle c-\rangle$ ($std(c-)$)	$\langle l\rangle$ ($std(l)$)	l_{max}
**Movielens**	172	788	1(0.12%)	2,162	0(0)	9.20 (11.21)	1(0)	NaN	0.429(0.367)	0(0)	2.878(0.568)	7
	142	237	23(9.7%)	84	8(9.52%)	3.24(2.80)	1.59(1.57)	0.220	0.2249(0.343)	0(0)	5.234(1.223)	13
**RYM**	1,576	33,439	10,872(32.51%)	1,134,866	364,073(32.08%)	44.55(57.66)	14.71(58.68)	-0.0042	0.739(0.350)	0(0)	3.309(0.899)	15
	102	5,151	0(0)	171,700	0(0)	101(0)	NaN	NaN	1(0)	NaN	1(0)	2
	79	183	144(78.69%)	156	126(80.77%)	2.79(1.97)	3.65(5.42)	0.627	0.459(0.461)	0(0)	3.091(0.632)	7
**Epinions**	999	1,758	1,393(79.24%)	184	67(36.41%)	2(1.71)	3.54(4.35)	-0.106	0.213(0.369)	0(0)	9.160(1.860)	26
	22	29	13(44.83%)	2	0(0)	1.88(1.73)	2.6(1.26)	0.157	0.161(0.334)	0(0)	2.707(0.535)	6
	21	21	0(0)	1	0(0)	2(1.38)	NaN	NaN	0.029(0.081)	NaN	3.415(0.675)	7
**Douban**	517	2,095	140(6.68%)	8373	65(0.78%)	9.35(12.40)	1.92(5.39)	-0.087	0.494(0.405)	0(0)	7.810(1.672)	24
	41	81	1(1.23%)	67	0(0)	4(4.21)	1(0)	NaN	0.437(0.405)	0(0)	2.892(0.745)	7
	27	40	0(0)	19	0(0)	2.96(2.21)	NaN	NaN	0.299(0.387)	NaN	3.232(0.702)	7

[1] Heider F. Attitudes and cognitive organization. *Journal of Psychology* **21(1)**, 107-112 (1946).

[2] LI G, et al. Towards design principles for optimal transport networks. *Phys. Rev. Lett.* **104**, 018701 (2010).

[3] Zeng, An, *et al.* Information filtering by similarity-preferential diffusion processes. *EPL* **105**, 58002 (2014).
